# Neuropharmacological Potential of *Gastrodia elata* Blume and Its Components

**DOI:** 10.1155/2015/309261

**Published:** 2015-10-12

**Authors:** Jung-Hee Jang, Yeonghoon Son, Seong Soo Kang, Chun-Sik Bae, Jong-Choon Kim, Sung-Ho Kim, Taekyun Shin, Changjong Moon

**Affiliations:** ^1^College of Veterinary Medicine and Animal Medical Institute, Chonnam National University, Gwangju 500-757, Republic of Korea; ^2^Cheongju Hamsoa Oriental Clinic, Cheongju 361-814, Republic of Korea; ^3^College of Veterinary Medicine, Jeju National University, Jeju 690-756, Republic of Korea

## Abstract

Research has been conducted in various fields in an attempt to develop new therapeutic agents for incurable neurodegenerative diseases. *Gastrodia elata* Blume (GE), a traditional herbal medicine, has been used in neurological disorders as an anticonvulsant, analgesic, and sedative medication. Several neurodegenerative models are characterized by oxidative stress and inflammation in the brain, which lead to cell death via multiple extracellular and intracellular signaling pathways. The blockade of certain signaling cascades may represent a compensatory therapy for injured brain tissue. Antioxidative and anti-inflammatory compounds isolated from natural resources have been investigated, as have various synthetic chemicals. Specifically, GE rhizome extract and its components have been shown to protect neuronal cells and recover brain function in various preclinical brain injury models by inhibiting oxidative stress and inflammatory responses. The present review discusses the neuroprotective potential of GE and its components and the related mechanisms; we also provide possible preventive and therapeutic strategies for neurodegenerative disorders using herbal resources.

## 1. Introduction

Incurable neurodegenerative disorders result in aplastic impairment of brain function. Many previous studies have identified the underlying etiology and pathogenesis of neurodegenerative disorders; however, current therapeutic strategies provide limited symptom relief or suppression of disease progression for incurable neurodegenerative disorders, including Alzheimer's disease (AD), Parkinson's disease (PD), stroke, and seizure. For example, AD is clinically treated using cholinesterase inhibitors, glutamate modulators [1], and antiamyloid *β* (A*β*) peptide agents to mitigate the symptoms and neurodegeneration [2]. Clinical therapies for the treatment of PD include L-3,4-dihydroxyphenylalanine (L-DOPA) for dopaminergic neuron degeneration and nondopaminergic drugs to alleviate nonmotor symptoms [3]. Thrombolytic agents have restricted use against acute ischemic stroke [4]. The development of new therapeutic agents that effectively treat and promote recovery in neurodegenerative diseases is urgently needed. Here, traditional herbal medicine is suggested to be a potential therapeutic approach as an alternative medicine for incurable neurodegenerative diseases.

A combination of several herbs is typically used clinically in traditional herbal medicine; the interactions among herbs following decoction of several medical herbs have synergistic effects that increase their efficacy and reduce possible adverse reactions by decreasing toxicity [5]. Because the pathogenesis of certain diseases involves multiple targets associated with different pathways, a complex prescription, such as decoction that includes multiple herbs, is an extremely beneficial therapeutic approach [6]. Thus, the discovery of new properties of traditional herbal medicines, such as herb-herb interactions and multiple targets, may provide a solution to the treatment of incurable neurodegenerative disorders. However, there are various limitations to the investigation of such complex prescriptions. For example, it is difficult to accurately evaluate the inherent efficacy of each herb in such complexes and to identify the target component involved in the disease mechanism and thus the treatment effect. Therefore, we must first study each individual component of traditional herbal medicines to determine the pharmacological mechanisms involved.

*Gastrodia elata* Blume (GE), which belongs to the Orchidaceae family, is a saprophyte that grows in the woods of East Asia. The dried rhizome (tuber) of this plant (tianma) is used as a traditional herbal medicine to treat neurological disorders such as vertigo, general paralysis, epilepsy, and tetanus. The GE rhizome has been used clinically as a complex prescription rather than as a single herb. For example,* Banxia Baishu Tianma Tang*, which is a decoction composed of GE rhizome and other herbs such as* Pinellia ternata* and* Atractylodes*, is prescribed to treat hypertension in East Asia [7, 8]. The effects of a complex prescription mixed together with GE rhizome and other herbs have been demonstrated in patients with Tourette's syndrome [9]. Ningdong granule (i.e., GE rhizome,* Codonopsis pilosula*,* Ophiopogon japonicus*, white peony root, Rhinocerotidae, oyster, earthworm, and licorice root) attenuated symptoms of Tourette's syndrome in children and returned abnormal levels of interleukin- (IL-) 12 and tumor necrosis factor- (TNF-) alpha in the serum to normal. In addition, many previous studies have attempted to elucidate the pharmacological effects of multiple herb decoctions that include GE rhizome to provide new therapeutic opportunities for neurodegenerative diseases [6, 10, 11]. Additionally, previous studies have investigated the pharmacokinetics of GE components. While higher relative bioavailability of gastrodin and parishin was obtained in rats after oral administration of GE rhizome powder at low doses, higher bioavailability was shown after administration of high doses of the GE rhizome aqueous extract [12]. Moreover, a detection technique was developed to determine the pharmacokinetics of gastrodin in rat blood, brain, and bile, and this technique might be a useful method for the determination of the metabolism of gastrodin [13]. However, further studies are necessary to elucidate the pharmacological and pharmacokinetic properties of GE and its components in the context of brain tissue injury.

**Figure 1:**
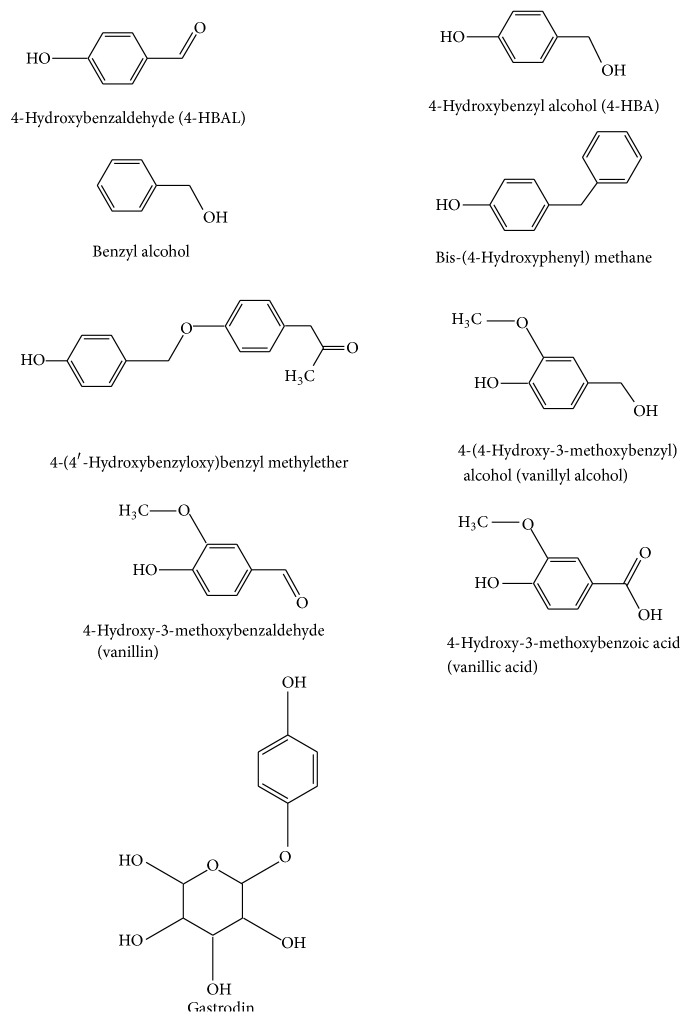
Chemical structure of representative* Gastrodia elata* Blume compounds.

Most of the previous studies have attempted to identify the biologically active components of the GE rhizome, and a variety of compounds have been isolated from the aqueous or methanol extracts of GE rhizome. Methanol extracts of GE rhizome reportedly exert neuroprotective and antioxidant effects [14]. One study isolated and identified 14 GE compounds using silica gel column chromatography and fractionated 8 phenolic components [15]: 4-hydroxybenzaldehyde (4-HBAL), 4-hydroxybenzyl alcohol (4-HBA), benzyl alcohol, bis-(4-hydroxyphenyl) methane, 4-(4′-hydroxybenzyloxy)benzyl methylether, 4-hydroxy-3-methoxybenzyl alcohol (vanillyl alcohol), 4-hydroxy-3-methoxybenzaldehyde (vanillin), and 4-hydroxy-3-methoxybenzoic acid (vanillic acid) (Figure 1). Among them, several GE compounds, including 4-HBAL, 4-HBA, benzyl alcohol, vanillyl alcohol, vanillin, and vanillic acid, are listed on the Everything Added to Food in the United States (EAFUS) database as Food and Drug Administration- (FDA-) approved food additives (http://www.accessdata.fda.gov/scripts/fcn/fcnnavigation.cfm?rpt=eafuslisting&displayAll=true). Several new compounds were recently isolated by various chromatography techniques; in total, 64 compounds were identified from GE rhizome, including parishin J and parishin K [16]. A previous study reported the metabolic profile of parishin in rat plasma and urine after administration of parishin to investigate the pharmacological effects [17]. However, among these components of GE, gastrodin, 4-HBAL, 4-HBA, vanillin, and vanillyl alcohol are the major active components in terms of their neuropharmacological properties [18]. Gastrodin, the main bioactive component of GE, has since been obtained via ethanol and aqueous extraction and is the phenolic glucoside of 4-HBA [19] (Figure 1). Another main bioactive component, vanillin, which can be isolated from methanol extracts, is an aromatic aldehyde that contains a hydroxyl group para to aldehyde (Figure 1). Vanillin may be effective as a new antiepileptic drug, as vanillin reportedly has effects on human epilepsy patients: 184 patients treated with vanillin monotherapy for 3 months showed improvement in a previous study [20]. In addition, vanillin is a potent anti-inflammatory agent that inhibits the generation of reactive oxygen species (ROS) [21]. Benzyl alcohol, 4-HBAL, and 4-HBA have anti-inflammatory effects via the inhibition of the activities of cyclooxygenase- (COX-) 1 and COX-2, and vanillyl alcohol significantly increases the radical-scavenging activity of DPPH [21].* In vivo* and* in vitro* experiments have demonstrated that GE and its components have various pharmacological actions that result in antioxidant, anti-inflammatory, and anticonvulsant effects [14, 20]. Here we report on the potential therapeutic potential of GE for the treatment of neurodegenerative disorders, including epilepsy, ischemia, AD, and PD.

In this review, we provide an overview of the efficacy of GE and its components in a variety of neurodegenerative models. We also discuss the possible mechanisms involved in ameliorating a broad range of brain disorders that lead to neuronal death.

## 2. Protective Effects of GE and Its Components in Neurodegenerative Disease Models

Several studies have revealed the effects of GE and its components on various* in vivo *and* in vitro *models of neurodegenerative disorders, such as epilepsy, ischemia, AD, and PD (Table 1).

### 2.1. Induced Seizure Model

Previous studies have demonstrated the anticonvulsant properties of GE in rodent models of seizure. Kainic acid (KA) is an excitatory agonist that induces limbic seizures and excitotoxicity in the hippocampus [22]. The anticonvulsant effect of GE rhizome extract has been documented in rodent KA models of temporal epilepsy [23–26]. The ether fraction of GE rhizome methanol extracts has anticonvulsant effects on this model, and histopathological findings have shown that treatment with ether fraction of GE rhizome extract attenuates KA-induced neuronal cell death in the hippocampal cornus ammonis (CA) 1 and 3 regions [25]. In addition, oral administration of GE rhizome ethanol extract significantly delayed the onset time of neurobehavioral change and reduced the number of seizure-like behaviors, such as wet dog shakes, paw tremor, and facial myoclonia, consistent with the reduced level of lipid peroxides in the rat brain [24]. Moreover, a previous study also demonstrated that GE rhizome aqueous extract reduced the epileptic attack durations by measuring behavioral observations, including wet dog shakes, paw tremor, and facial myoclonia [26]. Cocaine also reportedly induces seizures by inhibiting gamma aminobutyric acid (GABA)_A_ currents and enhancing dopamine and glutamate transmission [27, 28]. Treatment with GE rhizome methanol extract following cocaine administration delays the onset of neurobehavioral changes and shortens seizure duration [29]. Animals fed 4-HBAL from the ether fraction of GE rhizome methanol extracts exhibit less convulsant activities than rats who receive pentylenetetrazole (PTZ) treatment alone [30]. Based on previous studies, KA-, cocaine-, and PTZ-induced seizures may be suitable models for identifying the antiepileptic effects of GE and its components.

**Figure 2:**
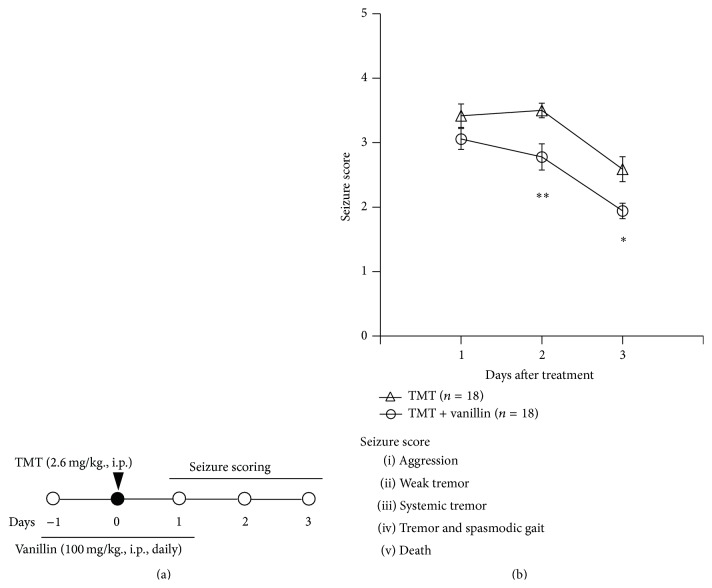
(a) Experimental scheme used to evaluate the effect of vanillin in a trimethyltin- (TMT-) induced seizure model. Mice received a single injection of TMT (2.6 mg/kg, intraperitoneal (i.p.)) and vanillin (100 mg/kg, i.p.) once daily for 3 days at −1 day, 0 days, and 1 day relative to TMT injection. Behavioral changes used to measure seizure activity were observed and scored 1, 2, and 3 days after TMT injection. (b) The anticonvulsant effect of vanillin against TMT-induced clinical seizure symptoms in C57BL/6 mice. Data are presented as means ± standard errors of the mean (SEM). ^*∗*^ *P* < 0.05, ^*∗∗*^ *P* < 0.01 versus TMT-treated group.

**Figure 3:**
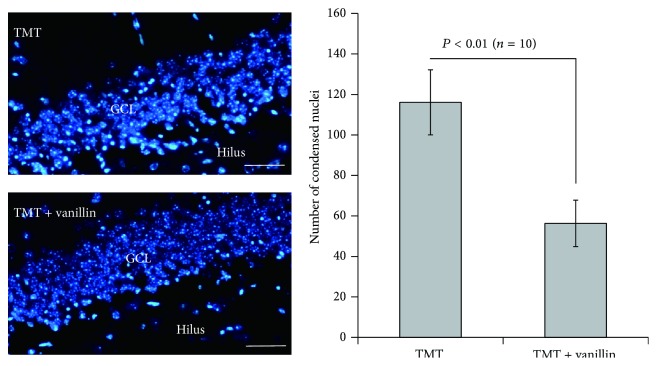
Inhibitory effect of vanillin on neuronal cell death in the granular cell layer after TMT injection. Mice received a single injection of TMT (2.6 mg/kg, intraperitoneal (i.p.)) and vanillin (100 mg/kg, i.p.) once daily for 3 days at −1 day, 0 days, and 1 day relative to TMT injection. Mice were sacrificed 3 days after TMT injection. Photomicrographs (left panels, 4′,6-diamidino-2-phenylindole (DAPI) staining) show that the increased amount of nuclear pyknosis induced by TMT treatment was significantly reduced by vanillin treatment. Semiquantitative analysis of neuronal cell death, performed by counting nuclear pyknosis, showed that vanillin suppressed neuronal cell death (*n* = 10 mice/group). Data are presented as means ± SEMs. Scale bars indicate 40 *μ*m.

The organotin compound trimethyltin (TMT) is a potent neurotoxicant whose effects are characterized by selective neuronal death in the limbic system, including the hippocampus [31]. In addition, we have performed experiments to identify the antiepileptic effects of vanillin, a GE component, in a TMT-induced seizure model. The vanillin used in this study was purchased from Sigma-Aldrich (Cat. number V1104) and was dissolved in 2% ethanol within phosphate-buffered saline (pH 7.4). As shown in Figure 2(a), mice received a single injection of TMT (2.6 mg/kg, intraperitoneal (i.p.)) and a daily injection of vanillin (100 mg/kg, i.p.) for 3 consecutive days. Seizure behaviors were examined 1–3 days after treatment, consistent with previous studies [32–35]. For statistical analysis, the data are reported as mean ± SEM and were analyzed by one-way analysis of variance (ANOVA) followed by the Student-Newman-Keuls* post hoc* test for multiple comparisons. In all analyses, *P* < 0.05 was taken to indicate statistical significance. Vanillin treatment significantly reduced seizure behaviors induced by TMT treatment (Figure 2(b)). In addition, during histological examination, we determined the amount of nuclear pyknosis in the granular cell layer (GCL) of the hippocampal dentate gyrus and observed a marked decrease in neuronal cell death, as in a previous study [33]. Semiquantitative analysis of nuclear pyknosis revealed that vanillin treatment significantly attenuated neuronal damage induced by TMT treatment (Figure 3). Thus, GE and its components may be potential therapeutic candidates for the treatment of epileptic seizures. Further study is necessary to identify the mechanisms of the anticonvulsant action by GE and a variety of its components and to detect components that are effective against human epilepsy.

### 2.2. Ischemia Model

Cerebral ischemia-induced neurological dysfunction is caused by secondary injury processes, including excitotoxicity, ionic imbalance, and ROS generation [36], which lead to neuronal cell death by inducing tissue infarction [37]. Thus, brain ischemia may share common mechanisms with neurodegenerative disorders. The neuroprotective properties of the GE or its components have been demonstrated in ischemic animal models. Studies have shown that vanillin, 4-HBAL, and 4-HBA significantly reduce neuronal cell death in the hippocampal CA1 region of Mongolian gerbils with transient global ischemia [38]. Further, the ether fraction of GE rhizome remarkably protects against hippocampal neuron damage in this model [39]. The phenolic glucoside gastrodin significantly decreased infarction volume and edema volume in the brain, improved neurological scores, and ameliorated cerebral injury in a rat ischemic model with middle cerebral artery occlusion (MCAO) [40]. In a previous study, involving the same ischemic model, gastrodin treatment before MCAO operation decreased the volume of cerebral infarction and the release of cerebral amino acids [41]. In addition, a previous* in vitro* study demonstrated that gastrodin pretreatment significantly increases neuronal survival in hypoxia-exposed rat cortical neurons [42]. These findings support the concept that GE or its components have protective effects against neuronal damage due to ischemia in* in vivo* and* in vitro* experiments, suggesting that GE and its components may act as potential preventive or therapeutic agents in human stroke.

### 2.3. AD and PD Models

AD is an important neurodegenerative disorder characterized by progressive cognitive impairment. A major pathological hallmark of AD is the accumulation of senile plaques composed of A*β* protein [43, 44]. Many previous studies have reported on the potential therapeutic properties of traditional herbs against AD. Among the compounds tested, GE is reportedly a promising candidate for use in protecting neuronal cells against AD pathogenesis [45, 46]. In rats injected with A*β*_25–35_ to model AD, chronic administration of powdered GE rhizome dissolved in water markedly reduced amyloid plaque deposition in the hippocampus and significantly improved impaired spatial memory in the Morris water maze test; these changes were consistent with the increased expression of choline acetyltransferase in the medial septum and hippocampus [47]. A previous study demonstrated the neuroprotective effect of GE rhizome chloroform extract* in vitro* using rat pheochromocytoma (PC12) cells incubated with A*β*_1–42_ [45]. In addition, methanol extract of GE rhizome and its pure components, gastrodin and 4-HBA, have been shown to have protective effects against A*β*-induced cell death in BV2 microglial cells, possibly through upregulation of glucose-regulated protein 78 (Grp78), an antiapoptotic endoplasmic reticulum (ER) stress protein related to protein-folding machinery [48].

Similar to AD, PD is one of the most common neurodegenerative disorders. It is characterized by a loss of dopaminergic neurons in the substantia nigra pars compacta, which leads to symptoms of rigidity, resting tremor, and bradykinesia [49]. The neurotoxin 1-methyl-4-phenyl-1,2,3,6-tetrahydropyridine (MPTP), which can be metabolized into 1-methyl-4-phenylpyridinium (MPP^+^), induces neuronal cell death and is widely used in animal models of PD [50, 51]. In a previous study that used the MPTP-induced PD mouse model, gastrodin had a neuroprotective effect, as demonstrated by reduced bradykinesia and motor impairment in the pole and rotarod tests, respectively [52]. In addition, gastrodin treatment significantly decreased the neuronal cell viability induced by MPP^+^ [52]. The protective effects of ethanol extract of GE rhizome or gastrodin against MPP^+^-induced neurotoxicity have also been demonstrated in SH-SY5Y cells by inhibiting oxidative and apoptotic signaling [53] and in dopaminergic cells by inducing heme oxygenase-1 (HO-1) expression [54]. In MN9D dopaminergic cells, vanillyl alcohol inhibits the cytotoxicity induced by MPP^+^ [55]. L-DOPA is a dopaminergic drug used to treat PD, but long-term L-DOPA treatment results in L-DOPA-induced dyskinesia (LID) [56]. Therefore, GE or its components have neuroprotective effects on* in vivo* and* in vitro* AD and PD models and may be potential preventive or therapeutic agents for human AD and PD.

## 3. Pharmacological Mechanisms of GE and Its Components

Several studies have attempted to clarify the pharmacological mechanisms of GE and its components in neurological disorders (Table 2).

### 3.1. Effects of GE and Its Components on Neurotransmission

GABA is the major inhibitory neurotransmitter in the central nervous system (CNS), and malfunction of its transmission may result in pathological conditions such as seizure, ischemia, and learning impairment. Previous studies have shown that GE and its components may confer neuroprotection by inhibiting the degradation of GABA and thus enhance GABA levels [30, 57, 67]. In rats, the decreased brain GABA content induced by PTZ treatment can be reversed by treatment with the ether fraction of the methanol extract of GE rhizome, suggesting that GE may have anticonvulsant activity [30]. In Mongolian gerbils, seizure severity can be attenuated by gastrodin treatment via inhibition of GABA-degrading enzymes, including GABA transaminase (GABA-T), succinic semialdehyde dehydrogenase (SSADH), and succinic semialdehyde reductase (SSAR), in the hippocampal regions [57]. Moreover, gastrodin has been found to regulate GABA neurotransmitter levels by inhibiting SSADH [68]. However, another study reported opposite findings regarding GABA-T levels after treatment with various GE components; in a transient global ischemia model, 4-HBA treatment increased GABA-T levels in the early stage of ischemia, which might have contributed to cell survival through the energy supply generated by rapid GABA degradation in neuronal cells [38]. This discrepancy among results regarding GABA-T levels may be related to differences in the pathogenesis of seizure and ischemia.

Methanol extract of GE rhizome delays seizure onset and shortens seizure duration in cocaine-induced convulsion by activating the GABA_A_ receptor [29]. A previous* in vivo* study showed that GE rhizome ethanol extract increased the total sleep time and reduced sleep latency in pentobarbital-treated mice, and an* in vitro* study also demonstrated an increased level of GABA_A_ receptors following treatment with GE rhizome ethanol extract [58]. In another study, the anxiolytic effects of 4-HBA and 4-HBAL were inhibited by WAY 100635, a serotonin (5-HT_1A_) receptor antagonist, and flumazenil, a GABA_A_ receptor antagonist, respectively [69]. These results indicate that GE extract and its components may be involved in the regulation of GABA_A_ receptor in neurological disorders. However, further studies are required to clarify the precise mechanisms underlying the effects of GE and its components on GABA-degradative enzymes and GABA receptors.

### 3.2. Effects of GE and Its Components on Oxidative Response

The level of ROS production is an important factor determining the severity of neurodegenerative disease, and enhancing antioxidant activity may be a possible mechanism involved in the neuroprotective effects of GE [70, 71]. In a transient global ischemia model, 4-HBA treatment decreased 8-hydroxy-2′-deoxyguanosine (8-OHdG) immunoreactivity, which is one of the major products of DNA oxidation [38]. In AD and PD* in vivo* and* in vitro* models, GE rhizome aqueous and ethanol extract ameliorated neurodegeneration by reducing oxidative stress, respectively [53, 60]. A previous study also demonstrated that the protective effect of GE rhizome aqueous extract in an AD model may be related to inhibition of apoptosis and upregulation of antioxidative enzymes, including catalase, superoxide dismutase (SOD), and glutathione peroxidase [60]. In addition, GE rhizome ethanol extract had a neuroprotective effect, as demonstrated by reductions in ROS production, Bax/Bcl-2 ratio, cleaved caspase-3, and PARP proteolysis induced by MPTP in a PD* in vitro* model with SH-SY5Y or MN9D cells, respectively [53, 55]. Gastrodin and vanillyl alcohol reduce ROS production in MPP^+^-induced neurotoxicity [54, 55]. Gastrodin may confer neuroprotection by enhancing the expression of antioxidant enzyme HO-1 via activation of the p38 mitogen-activated kinase (MAPK)/Nrf-2 pathway in human dopaminergic cells [54]. This compound has also been found to have antioxidative effects in a glutamate-induced injury model by measuring the levels of malondialdehyde, mitochondrial membrane potential, and superoxide dismutase [59]. In that study, gastrodin prevented glutamate-induced oxidative stress in PC12 cells by blocking [Ca^2+^]_I_ influx and inhibiting calmodulin-dependent kinase II (CaMKII) activation, apoptosis signal-regulating kinase 1 (ASK1), and p38 MAPK phosphorylation [59]. In a transient focal ischemia rat model, water extract of GE rhizome and 4-HBA treatment induced antioxidant gene transcription in the brain [61]. These studies have revealed parts of the mechanisms involved in the neuroprotective effects of GE and its components. Further studies of the mechanisms of action of other GE components are necessary.

### 3.3. Effects of GE and Its Components on Neuroinflammation

The role of the inflammatory response has been investigated in neurodegenerative disorders, including AD, PD, and epilepsy [72, 73]. Many studies have demonstrated that amelioration of inflammatory responses might be another possible mechanism by which GE and its components exert neuroprotective effects. In rat models of KA-induced epilepsy, treatment of GE rhizome ethanol extract reduces the number of activated microglial cells, with a concomitant decrease in neuronal nitric oxide synthase- (NOS-) stained cells [65]. In a rotenone-induced rat PD model, gastrodin inhibits microglial activation and inflammatory cytokines [64]. Depression-like behaviors can be reversed following gastrodin administration, possibly due to the inhibition of IL-1*β* expression, a proinflammatory cytokine [62]. In RAW264.7 macrophages, treatment of GE rhizome ethanol extract inhibits NO production and the expression of iNOS and COX-2 induced by lipopolysaccharide (LPS) [66]. In microglial BV-2 cells activated by LPS, GE rhizome ethanol extract inhibits inflammatory cytokines such as TNF-*α* and IL-1*β* and downregulates the c-Jun N-terminal kinase (JNK) and nuclear factor kappa-light-chain-enhancer of activated B cells (NF-*κ*B) signaling pathways [18]. Furthermore, gastrodin significantly reduces the protein and mRNA expression levels of iNOS, COX-2, TNF-*α*, IL-1*β*, and NF-*κ*B, which may be related to the inhibition of the NF-*κ*B signaling pathway and phosphorylation of MAPKs [63].

**Figure 4:**
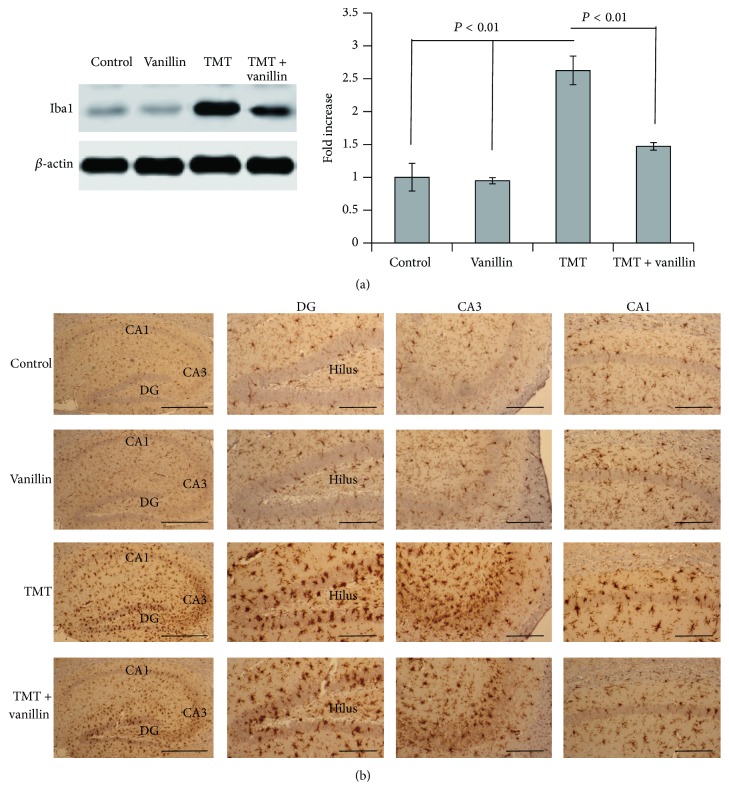
Inhibitory effect of vanillin on microglial activation in the mouse hippocampus after TMT treatment. Mice received a single injection of TMT (2.6 mg/kg, intraperitoneal (i.p.)) and vanillin (100 mg/kg, i.p.) once daily for 3 days at −1 day, 0 days, and 1 day relative to TMT injection. Mice were sacrificed 3 days after TMT injection. (a) Representative immunoblots show Iba1 (a marker of microglia) and *β*-actin expression in the mouse hippocampus. Bar graphs show that the increased Iba1 expression in the mouse hippocampus following TMT treatment was significantly ameliorated by vanillin treatment. Data are presented as means ± SEMs. (b) Photomicrographs show representative images of Iba1 expression in the dentate gyrus (DG), CA1, and CA3. The number of activated microglia, a hypertrophied form, following TMT treatment markedly decreased following vanillin treatment. Scale bars indicate 300 *μ*m in the left panels and 100 *μ*m in other panels.

Resident microglia transform into a phagocytic phenotype under stimuli such as cell death, accumulated debris, excess aberrant protein, or the presence of viral or bacterial pathogens. Furthermore, microglia serve important functions associated with inflammatory responses, cytotoxicity, repair, remodeling, and immunosuppression in brain injury and neurodegeneration [74]. We examined microglial activation and performed a biochemical analysis to determine whether vanillin has anti-inflammatory effects (Figure 4). The expression level of Iba1 immunoreactivity was measured by Western blotting, to semiquantitatively analyze the anti-inflammation effects of vanillin, as in previous studies [32, 34]. The expression level of Iba1 in the hippocampus after TMT administration was significantly increased, but the level was attenuated in the vanillin-treated group (Figure 4(a)). Iba1 immunoreactivity was assessed 3 days after TMT administration using immunohistochemical staining to investigate the histological and morphological changes in microglia in the hippocampus, as in previous studies [32, 34]. In the vehicle- and vanillin-treated controls, microglia showed few cell bodies within the GCL. In the TMT-treated group, activated microglia displaying a hypertrophied form with long, thickened, branching processes were prominent throughout the GCL (Figure 4(b)). However, in the TMT + vanillin-treated group, the density of activated microglia was reduced throughout the GCL, CA1, and CA3 (Figure 4(b)). Thus, our results confirmed that TMT-induced microglial activation was ameliorated by vanillin treatment in the mouse hippocampus after TMT treatment. Similar to GE rhizome ethanol extract and gastrodin [62, 65], vanillin, a GE component, may have anti-inflammatory effects by inhibiting microglial activation.

## 4. Conclusion

**Figure 5:**
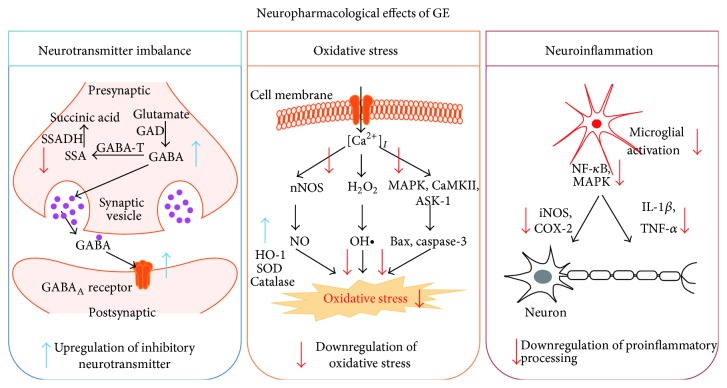
Schematic representation of the neuropharmacological effects of* Gastrodia elata* (GE). Multiple disease mechanisms, such as neurotransmitter imbalance, oxidative damage, and neuroinflammation, reportedly induce a variety of neurodegenerative disorders. GE has the potential to positively restore the neuronal cell damage in neurodegenerative diseases via the upregulation of inhibitory neurotransmitters and downregulation of oxidative stress and neuroinflammation. ASK-1: apoptosis signal-regulating kinase-1; CaMKII: Ca^2+^/calmodulin-dependent kinase II; COX-2: cyclooxygenase-2; GABA-T: gamma aminobutyric acid transaminase; GAD: glutamate decarboxylase; GE:* Gastrodia elata*; iNOS: inducible nitric oxide synthase; JNK: c-jun N-terminal kinases; MAPK: mitogen-activated protein kinase; NO: nitric oxide; SOD: superoxide dismutase; SSADH: succinic semialdehyde dehydrogenase.

Many patients suffer from incurable neurodegenerative disorders, but there are few therapeutic drugs for treating these diseases. The pathological mechanisms involved in neurodegenerative diseases are mediated by neurotransmitter imbalance, oxidative stress, and neuroinflammation; however, treatment efficacy is not satisfactory. Herbal decoctions including GE rhizome have been used in oriental medicine in East Asia to treat a variety of diseases. To reveal the active components within such herbal decoctions, numerous studies have investigated cellular and molecular mechanisms using GE and its components. In this review, we summarized the protective effects of GE against neurodegenerative disorders and proposed the underlying mechanisms of the neuropharmacological potential of GE and its components. These mechanisms may be related to the correction of neurotransmitter imbalance and inhibition of oxidative response and neuroinflammation (Figure 5). In addition, we confirmed that administration of vanillin, an active component of GE, ameliorates TMT-induced seizures, which may be related to the reduced neuronal death and microglial activation. Therefore, this review encourages the identification of specific GE components for use in possible preventive or therapeutic strategies for various neurodegenerative disorders and may also be helpful for the development of new treatments for incurable disorders.

## Figures and Tables

**Table 1:** Pathological models used to identify the effects of *Gastrodia elata* (GE) and its components on neurodegenerative disorders.

Model	Inducer	Extracts/components	Dose/route/regimen	Animal or cell	Major finding	Reference
Seizure	Cocaine	GE rhizome—methanol extract	500 or 1000 mg/kg, p.o., 5 times every 12 h before cocaine treatment	C57BL/6J mice	Seizure onset time ↑ Seizure duration ↓	[29]
	KA	EFME of GE	200 or 500 mg/kg, p.o., 14 days before and 4 days after KA injection	ICR mice	Onset time of neurobehavioral change ↑ Severity of convulsions ↓ Hippocampal neuronal damage ↓	[25]
	KA	GE rhizome—ethanol extract	0.5 or 1.0 g/kg, p.o., 30 min before KA injection	SD rats	Seizure onset time ↑ Seizure-like behavior ↓	[24]
	KA	GE rhizome—aqueous extract	0.5 or 1.0 g/kg, p.o., 1 week before or 2 weeks after KA injection	SD rats	Three types of seizure (wet dog shakes, paw tremor, and facial myoclonia) ↓	[26]
	PTZ	EFME of GE rhizome	500 mg/kg, p.o., for 10 days	SD rats	Seizure recovery time ↓ Seizure severity ↓	[30]

Ischemia	Hypoxia	Gastrodin	25–100 *μ*g/mL	Rat cortical neurons	Neuronal survival ↑	[42]
	MCAO	Gastrodin	50 mg/kg, i.p., 10 min before MCAO	SD rats	Infarct volume ↓ Cerebral injury ↓ Amino acids ↑	[41]
	MCAO	Gastrodin	50 or 100 mg/kg, i.p., at the onset of MCAO	SD rats	Infarct volume ↓ Edema volume ↓	[40]
	Transient global ischemia	EFME of GE rhizome	200 or 500 mg/kg, p.o., 14 days before brain ischemia	Mongolian gerbils	Neuronal damage in hippocampal CA1 ↓	[39]
	Transient global ischemia	Vanillin, 4-HBAL, and 4-HBA	40 mg/kg, i.p., 30 min before and after ischemia	Mongolian gerbils	Neuronal survival in hippocampal CA1 ↑	[38]

Alzheimer's disease	A*β* (1–42)	GE rhizome—chloroform extract	20 *μ*g/mL, for 24 h	PC12 and primary neuronal cells	Neuronal cell protection ↑	[45]
	A*β* (1–42)	GE rhizome—methanol extract, gastrodin, and 4-HBA	10 *μ*g/mL, for 48 h	BV2 mouse microglial cell	Cell viability ↑	[48]
	A*β* (25–35)	GE rhizome powder	500 or 1000 mg/kg, p.o., for 52 days	Wistar rats	Amyloid deposits ↓ Spatial memory ↑ Choline acetyltransferase ↑	[47]

Parkinson's disease	MPP^+^	Gastrodin	1, 5, and 25 *μ*M, 4 h prior to MPP^+^ exposure	SH-SY5Y cells	Cell viability ↑ Oxidative stress ↓	[54]
	MPP^+^	GE rhizome—ethanol extract	10, 100, and 200 *μ*g/mL, 4 h prior to MPP^+^ exposure	SH-SY5Y cells	Cell viability ↑ Cytotoxicity ↓	[53]
	MPP^+^	GE rhizome—ethanol extract Vanillyl alcohol	10, 100, and 200 *μ*g/mL 1, 10, and 100 *μ*M	MN9D cells	Cell viability ↑ Cytotoxicity ↓	[55]
	MPTP, MPP^+^	Gastrodin	10, 30, and 60 mg/kg, p.o., for 15 days 1, 5, and 25 *μ*M, 4 h prior to MPP^+^ exposure	C57BL/6 mice, SH-SY5Y cells	Bradykinesia ↓ Motor impairment ↓ Cell viability ↑	[52]

4-HBA: 4-hydroxybenzyl alcohol; 4-HBAL: 4-hydroxybenzaldehyde; 6-OHDA: 6-hydroxydopamine; EFME: ether fraction of methanol extract; GE: *Gastrodia elata*; ICR: Institute of Cancer Research; KA: kainic acid; L-DOPA: L-3,4-dihydroxyphenylalanine; MCAO: middle cerebral artery occlusion; MPP^+^: 1-methyl-4-phenylpyridinium; MPTP: 1-methyl-4-phenyl-1,2,3,6-tetrahydropyridine; PC: pheochromocytoma; PTZ: pentylenetetrazole; SD: Sprague Dawley.

**Table 2:** Pharmacological effects of GE and its components on neurodegeneration.

Target pathway	Inducer(s)	Extracts/components	Dose/route/regimen	Animals or cells	Histological and biochemical evaluation	References

Neurotransmission	PTZ	EFME of GE rhizome	500 mg/kg, p.o., for 10 days	SD rats	Recovery of brain GABA contents	[30]

Neurotransmission	Stroking of the back	Gastrodin	60 mg/kg, p.o., for 1 week	Mongolian gerbils	Decreased GABA-T immunoreactivity Decreased SSADH, SSAR immunoreactivity	[57]

Neurotransmission	Cocaine	GE rhizome—methanol extract	500 or 1000 mg/kg, p.o., 5 times every 12 h before cocaine treatment	C57BL/6J mice	Attenuation of cocaine-induced seizure via GABA_A_, but not GABA_B_ receptor activation	[29]

Neurotransmission	Pentobarbital	GE rhizome—ethanol extract	25, 50, and 100 mg/kg, p.o., 1 h before pentobarbital injection	ICR mice, primary culture of cerebellar granule cells	Prolonged total sleep time Reduced sleep latency Increased GAD and GABA_A_ receptor subtype expression	[58]

Neurotransmission and antioxidation	Transient global ischemia	Vanillin, 4-HBAL, and 4-HBA	40 mg/kg, s.c., 30 min before and 30 min, 3, 6, 9, and 24 h after ischemia 0.016, 0.08, 0.4, 2, and 10 *μ*M, for 2 h	Mongolian gerbils, PC12 cells	Downregulated 8-OHdG immunoreactivity Increased GABA-T in the early stage after ischemia Antioxidant activity on lipid peroxidation	[38]

Antioxidation	Glutamate	Gastrodin	0.1, 1, and 10 *μ*M	PC12 cells	Inhibited ROS production Inhibited MDA, MMP, and SOD levels Blocked glutamate-induced [Ca^2+^]_I_ influx Blocked CaMKII, ASK-1, and phosphorylation of MAPK	[59]

Antioxidation	MPP+	Gastrodin	1, 5, and 25 *μ*M, 4 h prior to MPP^+^ exposure	SH-SY5Y cells	Decreased ROS production Induced HO-1 expression through p38 MAPK/Nrf2 signaling pathway	[54]

Antioxidation	MPP^+^	GE rhizome—ethanol extract	10, 100, and 200 *μ*g/mL, 4 h prior to MPP^+^ exposure	SH-SY5Y cells	Inhibited ROS production Inhibited Bax/Bcl-2 ratio, cleaved caspase-3, and PARP proteolysis	[53]

Antioxidation	MPP^+^	GE rhizome—ethanol extract Vanillyl alcohol	10, 100, and 200 *μ*g/mL 1, 10, and 100 *μ*M	MN9D cells	Inhibited ROS production Inhibited Bax/Bcl-2 ratio, cleaved caspase-3, and PARP proteolysis	[55]

Antioxidation	A*β* (25–35)	GE rhizome—aqueous extract	20 *μ*M, for 48 h	PC12 cells	Decreased ROS production Upregulated enzymatic activities of catalase, SOD, and glutathione peroxidase	[60]

Antioxidation	MCAO	GE rhizome—aqueous extract 4-HBA	500 mg/kg, i.p. 25 mg/kg, i.p., 3 days prior to MCAO	SD rats	Increased levels of genes related to antioxidant system (protein disulfide isomerase and 1-Cys peroxiredoxin)	[61]

Anti-inflammation	CUS	Gastrodin	50, 100, and 200 mg/kg, i.p., for 14 days 5, 10, 20, and 50 *μ*g/mL	SD rats Primary hippocampal cells	Upregulated neural stem cell proliferation Reduced NF-*κ*B and IL-1*β*	[62]

Anti-inflammation	LPS	Gastrodin	30, 40, and 60 *μ*M, 1 h prior to LPS exposure	BV-2 cells	Decreased levels of proinflammatory enzymes (iNOS, COX-2) and proinflammatory cytokines (TNF-*α*, IL-1*β*) Reduced phosphorylation of ERK1/2, JNK, p38 MAPK, and CREB	[63]

Anti-inflammation	Rotenone	Gastrodin	0.2 g/kg, p.o.	Wistar rats	Suppressed microglial activation Reduced IL-1*β* expression	[64]

Anti-inflammation	KA	GE rhizome—ethanol extract	0.5, 1 g/kg, p.o., 30 min prior to KA injection	SD rats	Decreased microglial activation (ED1) Reduced nNOS activation	[65]

Anti-inflammation and antioxidation	LPS	GE rhizome—ethanol extract	0.25, 0.5, and 1 *μ*g/mL, for 24 h	RAW264.7 cells	Inhibited NO production Reduced iNOS and COX-2 expression	[66]

Anti-inflammation and antioxidation	LPS	GE rhizome—ethanol extract 4-HBA	1, 10, and 100 *μ*g/mL 50, 100, and 200 nM	BV-2 cells	Inhibited JNK and NF-*κ*B signaling pathways Inhibited NO and iNOS	[18]

4-HBA: 4-hydroxybenzyl alcohol; 4-HBAL: 4-hydroxybenzaldehyde; 8-OHdG: 8-hydroxy-2′-deoxyguanosine; ASK-1: apoptosis signal-regulating kinase-1; CaMKII: Ca^2+^/calmodulin-dependent kinase II; COX-2: cyclooxygenase-2; CUS: chronic unpredictable stress; EFME: ether fraction of methanol extract; ERK: extracellular signal-regulated kinase; GABA-T: gamma aminobutyric acid transaminase; GAD: glutamate decarboxylase; GE: *Gastrodia elata*; GREE: *Gastrodia elata *rhizome ethanol extract; iNOS: inducible nitric oxide synthase; JNK: c-jun N-terminal kinases; KA: kainic acid; LPS: lipopolysaccharide; MAPK: mitogen-activated protein kinase; MCAO: middle cerebral artery occlusion; MDA: malondialdehyde; MMP: mitochondrial membrane potential; nNOS: neuronal nitric oxide synthase; NO: nitric oxide; PTZ: pentylenetetrazole; ROS: reactive oxygen species; SOD: superoxide dismutase; SSADH: succinic semialdehyde dehydrogenase; SSAR: succinic semialdehyde reductase.

## References

[1] Knopman D. S. (2006). Current treatment of mild cognitive impairment and Alzheimer's disease. *Current Neurology and Neuroscience Reports*.

[2] Yamada K., Nabeshima T. (2000). Animal models of Alzheimer's disease and evaluation of anti-dementia drugs. *Pharmacology & Therapeutics*.

[3] Schapira A. H. V., Bezard E., Brotchie J. (2006). Novel pharmacological targets for the treatment of Parkinson's disease. *Nature Reviews Drug Discovery*.

[4] Davis S., Lees K., Donnan G. (2006). Treating the acute stroke patient as an emergency: current practices and future opportunities. *International Journal of Clinical Practice*.

[5] Shi L., Tang X., Dang X. (2015). Investigating herb-herb interactions: the potential attenuated toxicity mechanism of the combined use of *Glycyrrhizae radix et rhizoma* (Gancao) and Sophorae flavescentis radix (Kushen). *Journal of Ethnopharmacology*.

[6] Chik S. C. C., Or T. C. T., Luo D., Yang C. L. H., Lau A. S. Y. (2013). Pharmacological effects of active compounds on neurodegenerative disease with gastrodia and uncaria decoction, a commonly used poststroke decoction. *The Scientific World Journal*.

[7] Xiong X., Yang X., Liu Y., Zhang Y., Wang P., Wang J. (2013). Chinese herbal formulas for treating hypertension in traditional Chinese medicine: perspective of modern science. *Hypertension Research*.

[8] Jiang J. Y., Wang X. Z., Luo S. S. (2010). Effect of banxia baizhu tianma decoction on the left ventricular hypertrophy of hypertrophied myocardium in spontaneously hypertensive rat. *Zhongguo Zhong Xi Yi Jie He Za Zhi Zhongguo Zhongxiyi Jiehe Zazhi*.

[9] Tang H.-X., Li A.-Y., Li J.-J., Hou G.-S., Zhang F. (2014). Effect of Ningdong Granule on the levels of IL-12 and TNF-alpha in children patients with Tourette's syndrome. *Zhongguo Zhong Xi Yi Jie He Za Zhi Zhongguo Zhongxiyi Jiehe Zazhi*.

[10] Chen P.-J., Sheen L.-Y. (2011). *Gastrodiae rhizoma* (tiān má): a review of biological activity and antidepressant mechanisms. *Journal of Traditional and Complementary Medicine*.

[11] Liu J., Mori A. (1992). Antioxidant and free radical scavenging activities of *Gastrodia elata* Bl. and *Uncaria rhynchophylla* (Miq.) Jacks. *Neuropharmacology*.

[12] Zhao Y., Gong X.-J., Zhou X., Kang Z.-J. (2014). Relative bioavailability of gastrodin and parishin from extract and powder of *Gastrodiae rhizoma* in rat. *Journal of Pharmaceutical and Biomedical Analysis*.

[13] Lin L.-C., Chen Y.-F., Lee W.-C., Wu Y.-T., Tsai T.-H. (2008). Pharmacokinetics of gastrodin and its metabolite *p*-hydroxybenzyl alcohol in rat blood, brain and bile by microdialysis coupled to LC-MS/MS. *Journal of Pharmaceutical and Biomedical Analysis*.

[14] Jung T.-Y., Suh S.-I., Lee H. (2007). Protective effects of several components of *Gastrodia elata* on lipid peroxidation in gerbil brain homogenates. *Phytotherapy Research*.

[15] Duan X.-H., Li Z.-L., Yang D.-S., Zhang F.-L., Lin Q., Dai R. (2013). Study on the chemical constituents of *Gastrodia elata*. *Zhong Yao Cai*.

[16] Li Z., Wang Y., Ouyang H. (2015). A novel dereplication strategy for the identification of two new trace compounds in the extract of Gastrodia elata using UHPLC/Q-TOF-MS/MS. *Journal of Chromatography B: Analytical Technologies in the Biomedical and Life Sciences*.

[17] Tang C., Wang L., Li J., Liu X., Cheng M., Xiao H. (2015). Analysis of the metabolic profile of parishin by ultra-performance liquid chromatography/quadrupole-time of flight mass spectrometry. *Biomedical Chromatography*.

[18] Kim B.-W., Koppula S., Kim J.-W. (2012). Modulation of LPS-stimulated neuroinflammation in BV-2 microglia by *Gastrodia elata*: 4-hydroxybenzyl alcohol is the bioactive candidate. *Journal of Ethnopharmacology*.

[19] Li H.-B., Chen F. (2004). Preparative isolation and purification of gastrodin from the Chinese medicinal plant *Gastrodia elata* by high-speed counter-current chromatography. *Journal of Chromatography A*.

[20] Ojemann L. M., Nelson W. L., Shin D. S., Rowe A. O., Buchanan R. A. (2006). Tian ma, an ancient Chinese herb, offers new options for the treatment of epilepsy and other conditions. *Epilepsy & Behavior*.

[21] Lee J. Y., Jang Y. W., Kang H. S., Moon H., Sim S. S., Kim C. J. (2006). Anti-inflammatory action of phenolic compounds from *Gastrodia elata* root. *Archives of Pharmacal Research*.

[22] Young A. B., Fagg G. E. (1990). Excitatory amino acid receptors in the brain: membrane binding and receptor autoradiographic approaches. *Trends in Pharmacological Sciences*.

[23] Hsieh C.-L., Tang N.-Y., Chiang S.-Y., Hsieh C.-T., Jaung-Geng L. (1999). Anticonvulsive and free radical scavenging actions of two herbs, Uncaria rhynchophylla (MIQ) Jack and *Gastrodia elata* Bl., in kainic acid-treated rats. *Life Sciences*.

[24] Hsieh C.-L., Chiang S.-Y., Cheng K.-S. (2001). Anticonvulsive and free radical scavenging activities of *Gastrodia elata* Bl. in kainic acid-treated rats. *The American Journal of Chinese Medicine*.

[25] Kim H.-J., Moon K.-D., Oh S.-Y., Kim S.-P., Lee S.-R. (2001). Ether fraction of methanol extracts of *Gastrodia elata*, a traditional medicinal herb, protects against kainic acid-induced neuronal damage in the mouse hippocampus. *Neuroscience Letters*.

[26] Hsieh C.-L., Lin J.-J., Chiang S.-Y. (2007). *Gastrodia elata* modulated activator protein 1 via c-Jun N-terminal kinase signaling pathway in kainic acid-induced epilepsy in rats. *Journal of Ethnopharmacology*.

[27] Ushijima I., Kobayashi T., Suetsugi M., Watanabe K., Yamada M., Yamaguchi K. (1998). Cocaine: evidence for NMDA-, beta-carboline- and dopaminergic-mediated seizures in mice. *Brain Research*.

[28] Ye J.-H., Ren J. (2006). Cocaine inhibition of GABA_A_ current: role of dephosphorylation. *Critical Reviews in Neurobiology*.

[29] Shin E.-J., Bach J.-H., Nguyen T.-T. L. (2011). *Gastrodia elata* Bl attenuates cocaine-induced conditioned place preference and convulsion, but not behavioral sensitization in mice: importance of GABAA receptors. *Current Neuropharmacology*.

[30] Ha J.-H., Lee D.-U., Lee J.-T. (2000). 4-Hydroxybenzaldehyde from *Gastrodia elata* B1. is active in the antioxidation and GABAergic neuromodulation of the rat brain. *Journal of Ethnopharmacology*.

[31] Besser R., Krämer G., Thümler R., Bohl J., Gutmann L., Hopf H. C. (1987). Acute trimethyltin limbic-cerebellar syndrome. *Neurology*.

[32] Yang M., Kim J., Kim T. (2012). Possible involvement of galectin-3 in microglial activation in the hippocampus with trimethyltin treatment. *Neurochemistry International*.

[33] Kim J., Yang M., Kim S.-H. (2013). Possible role of the glycogen synthase kinase-3 signaling pathway in trimethyltin-induced hippocampal neurodegeneration in mice. *PLoS ONE*.

[34] Kim J., Yang M., Son Y. (2014). Glial activation with concurrent up-regulation of inflammatory mediators in trimethyltin-induced neurotoxicity in mice. *Acta Histochemica*.

[35] Lee S., Yang M., Kim J. (2014). Nestin expression and glial response in the hippocampus of mice after trimethyltin treatment. *Acta Histochemica*.

[36] Iadecola C. (1997). Bright and dark sides of nitric oxide in ischemic brain injury. *Trends in Neurosciences*.

[37] Endres M., Biniszkiewicz D., Sobol R. W. (2004). Increased postischemic brain injury in mice deficient in uracil-DNA glycosylase. *The Journal of Clinical Investigation*.

[38] Kim H. J., Hwang I. K., Won M. H. (2007). Vanillin, 4-hydroxybenzyl aldehyde and 4-hydroxybenzyl alcohol prevent hippocampal CA1 cell death following global ischemia. *Brain Research*.

[39] Kim H.-J., Lee S.-R., Moon K.-D. (2003). Ether fraction of methanol extracts of *Gastrodia elata*, medicinal herb protects against neuronal cell damage after transient global ischemia in gerbils. *Phytotherapy Research*.

[40] Zeng X., Zhang S., Zhang L., Zhang K., Zheng X. (2006). A study of the neuroprotective effect of the phenolic glucoside gastrodin during cerebral ischemia in vivo and in vitro. *Planta Medica*.

[41] Bie X., Chen Y., Han J., Dai H., Wan H., Zhao T. (2007). Effects of gastrodin on amino acids after cerebral ischemia-reperfusion injury in rat striatum. *Asia Pacific Journal of Clinical Nutrition*.

[42] Xu X., Lu Y., Bie X. (2007). Protective effects of gastrodin on hypoxia-induced toxicity in primary cultures of rat cortical neurons. *Planta Medica*.

[43] Findeis M. A. (2000). Approaches to discovery and characterization of inhibitors of amyloid *β*-peptide polymerization. *Biochimica et Biophysica Acta*.

[44] Postuma R. B., He W., Nunan J. (2000). Substrate-bound beta-amyloid peptides inhibit cell adhesion and neurite outgrowth in primary neuronal cultures. *Journal of Neurochemistry*.

[45] Kim D. S. H. L., Kim J.-Y., Han Y. S. (2007). Alzheimer's disease drug discovery from herbs: neuroprotectivity from beta-amyloid (1-42) insult. *Journal of Alternative and Complementary Medicine*.

[46] Su Y., Wang Q., Wang C., Chan K., Sun Y., Kuang H. (2014). The treatment of Alzheimer's disease using Chinese Medicinal Plants: from disease models to potential clinical applications. *Journal of Ethnopharmacology*.

[47] Huang G.-B., Zhao T., Muna S. S. (2013). Therapeutic potential of *Gastrodia elata* blume for the treatment of alzheimer's disease. *Neural Regeneration Research*.

[48] Lee G.-H., Kim H.-R., Han S.-Y. (2012). *Gastrodia elata* blume and its pure compounds protect BV-2 microglial-derived cell lines against *β*-amyloid: the involvement of GRP78 and CHOP. *Biological Research*.

[49] Dauer W., Przedborski S. (2003). Parkinson's disease: mechanisms and models. *Neuron*.

[50] Bloem B. R., Irwin I., Buruma O. J. S. (1990). The MPTP model: versatile contributions to the treatment of idiopathic Parkinson's disease. *Journal of the Neurological Sciences*.

[51] Tipton K. F., Singer T. P. (1993). Advances in our understanding of the mechanisms of the neurotoxicity of MPTP and related compounds. *Journal of Neurochemistry*.

[52] Kumar H., Kim I.-S., More S. V., Kim B.-W., Bahk Y.-Y., Choi D.-K. (2013). Gastrodin protects apoptotic dopaminergic neurons in a toxin-induced Parkinson's disease model. *Evidence-Based Complementary and Alternative Medicine*.

[53] An H., Kim I. S., Koppula S. (2010). Protective effects of *Gastrodia elata* Blume on MPP^+^-induced cytotoxicity in human dopaminergic SH-SY5Y cells. *Journal of Ethnopharmacology*.

[54] Jiang G., Hu Y., Liu L., Cai J., Peng C., Li Q. (2014). Gastrodin protects against MPP^+^-induced oxidative stress by up regulates heme oxygenase-1 expression through p38 MAPK/Nrf2 pathway in human dopaminergic cells. *Neurochemistry International*.

[55] Kim I. S., Choi D.-K., Jung H. J. (2011). Neuroprotective effects of vanillyl alcohol in gastrodia elata blume through suppression of oxidative stress and anti-apoptotic activity in toxin-induced dopaminergic MN9D cells. *Molecules*.

[56] Fabbrini G., Brotchie J. M., Grandas F., Nomoto M., Goetz C. G. (2007). Levodopa-induced dyskinesias. *Movement Disorders*.

[57] An S.-J., Park S.-K., Hwang I. K. (2003). Gastrodin decreases immunoreactivities of *γ*-aminobutyric acid shunt enzymes in the hippocampus of seizure-sensitive gerbils. *Journal of Neuroscience Research*.

[58] Choi J. J., Oh E.-H., Lee M. K., Chung Y. B., Hong J. T., Oh K.-W. (2014). Gastrodiae Rhizoma ethanol extract enhances pentobarbital-induced sleeping behaviors and rapid eye movement sleep via the activation of GABA_A_-ergic transmission in rodents. *Evidence-Based Complementary and Alternative Medicine*.

[59] Jiang G., Wu H., Hu Y., Li J., Li Q. (2014). Gastrodin inhibits glutamate-induced apoptosis of PC12 cells via inhibition of CaMKII/ASK-1/p38 MAPK/p53 signaling cascade. *Cellular and Molecular Neurobiology*.

[60] Ng C.-F., Ko C.-H., Koon C.-M. (2013). The aqueous extract of rhizome of *Gastrodia elata* protected *Drosophila* and PC12 cells against beta-amyloid-induced neurotoxicity. *Evidence-Based Complementary and Alternative Medicine*.

[61] Yu S. J., Kim J. R., Lee C. K. (2005). Gastrodia elata blume and an active component, *p*-hydroxybenzyl alcohol reduce focal ischemic brain injury through antioxidant related gene expressions. *Biological and Pharmaceutical Bulletin*.

[62] Wang H., Zhang R., Qiao Y. (2014). Gastrodin ameliorates depression-like behaviors and up-regulates proliferation of hippocampal-derived neural stem cells in rats: involvement of its anti-inflammatory action. *Behavioural Brain Research*.

[63] Dai J.-N., Zong Y., Zhong L.-M. (2011). Gastrodin inhibits expression of inducible no synthase, cyclooxygenase-2 and proinflammatory cytokines in cultured LPS-stimulated microglia via MAPK pathways. *PLoS ONE*.

[64] Li C., Chen X., Zhang N., Song Y., Mu Y. (2012). Gastrodin inhibits neuroinflammation in rotenone-induced Parkinson's disease model rats. *Neural Regeneration Research*.

[65] Hsieh C.-L., Chen C.-L., Tang N.-Y. (2005). *Gastrodia elata* BL mediates the suppression of nNOS and microglia activation to protect against neuronal damage in kainic acid-treated rats. *The American Journal of Chinese Medicine*.

[66] Ahn E.-K., Jeon H.-J., Lim E.-J., Jung H.-J., Park E.-H. (2007). Anti-inflammatory and anti-angiogenic activities of *Gastrodia elata* Blume. *Journal of Ethnopharmacology*.

[67] Choi J.-H., Lee D.-U. (2006). A new citryl glycoside from *Gastrodia elata* and its inhibitory activity on GABA transaminase. *Chemical & Pharmaceutical Bulletin*.

[68] Baek N.-I., Choi S. Y., Park J. K. (1999). Isolation and identification of succinic semialdehyde dehydrogenase inhibitory compound from the rhizome of *Gastrodia elata* blume. *Archives of Pharmacal Research*.

[69] Jung J. W., Yoon B. H., Oh H. R. (2006). Anxiolytic-like effects of *Gastrodia elata* and its phenolic constituents in mice. *Biological and Pharmaceutical Bulletin*.

[70] Szeto H. H. (2006). Mitochondria-targeted peptide antioxidants: novel neuroprotective agents. *The AAPS Journal*.

[71] Pieczenik S. R., Neustadt J. (2007). Mitochondrial dysfunction and molecular pathways of disease. *Experimental and Molecular Pathology*.

[72] Liu B., Hong J.-S. (2003). Role of microglia in inflammation-mediated neurodegenerative diseases: mechanisms and strategies for therapeutic intervention. *The Journal of Pharmacology and Experimental Therapeutics*.

[73] Vezzani A., Granata T. (2005). Brain inflammation in epilepsy: experimental and clinical evidence. *Epilepsia*.

[74] Sierra A., Abiega O., Shahraz A., Neumann H. (2013). Janus-faced microglia: beneficial and detrimental consequences of microglial phagocytosis. *Frontiers in Cellular Neuroscience*.

